# DLAT promotes triple-negative breast cancer progression via YAP1 activation

**DOI:** 10.1080/15384047.2024.2421578

**Published:** 2024-10-26

**Authors:** Diya Liu, Xuehui Wang, Fengyuan Qian, Danrong Ye, Xiaochong Deng, Lin Fang

**Affiliations:** a Department of Thyroid and Breast Surgery, Shanghai Tenth People’s Hospital, School of Medicine, Tongji University, Shanghai, China; b Department of Breast Surgery, The First Affiliated Hospital of Wenzhou Medical University, Wenzhou, China; c Department of Breast Surgery, Guizhou Provincial People’s Hospital, Guiyang, China

**Keywords:** Breast cancer, DLAT, triple-negative breast cancer, tumor progression, YAP1

## Abstract

**Background:**

Breast cancer (BC) is the most prevalent malignant tumor in women globally. Triple-negative breast cancer (TNBC) represents the most malignant and invasive subtype of BC. New therapeutic targets are urgently needed for TNBC owing to its receptor expression characteristics, which render it insensitive to traditional targeted and endocrine therapies for BC. The role and mechanisms of dihydrolipoamide S-acetyltransferase (DLAT) as a crucial molecule in glycometabolism and cuproptosis-related biological processes in tumors remain to be explored.

**Methods:**

DLAT expression was investigated using bioinformatics methods and quantitative real-time polymerase chain reaction. Subsequently, the MTT assay, colony formation assay, and migration-invasion assay were performed to validate the effect of DLAT on TNBC cell viability, proliferation, and migration. Cytoplasmic-nuclear separation experiments, western blot analysis, and co-immunoprecipitation assays were performed to elucidate the underlying molecular mechanisms.

**Results:**

This study revealed a robust correlation between elevated DLAT expression in BC and unfavorable prognosis in patients, with higher expression of DLAT compared to other subtypes in TNBC. Functional cytology experiments indicated that DLAT plays a tumor-promoting role in TNBC. Mechanistic studies showed that DLAT directly interacts with YAP1, leading to the dephosphorylation and activation of YAP1 and its increased nuclear translocation, thereby transcriptionally activating and regulating downstream oncogenes, promoting the malignant phenotype of TNBC. Rescue experiments indicated that DLAT promotes the malignant behavior of TNBC through a YAP1-dependent pathway.

**Conclusions:**

Our research unveiled the significant involvement of DLAT in TNBC, along with the potential for modulating DLAT/YAP1 activity as a targeted treatment strategy for TNBC.

## 1. Introduction

Breast cancer (BC) is a significant determinant impacting women’s health. Currently, it has emerged as the cancer category exhibiting the highest rate of newly diagnosed cases globally, as well as the malignant neoplasm with the highest occurrence rate among women.^1^ One of the most dangerous subtypes of BC is triple-negative breast cancer (TNBC), which is characterized by the absence of the estrogen receptor (ER), progesterone receptor (PR), and human epidermal growth factor receptor 2 (HER2). This subtype constitutes approximately 15–20% of all BC cases.^2^ TNBC is widely considered highly malignant and metastatic and exhibits the worst prognosis among BC subtypes, accounting for 25% of all BC-related mortality.^3^ Employing endocrine therapy and targeted therapy in TNBC poses challenges. The prevailing therapeutic modalities primarily consist of conventional radiation and chemotherapy; however, their effectiveness is significantly constrained.^4^ Several recent studies have demonstrated that investigating the specific mechanisms underlying the progression^5,6^ or metastasis^7^ of TNBC is instrumental in identifying efficacious targets. At present, there is a significant emphasis on investigating therapeutic targets for TNBC, aiming to identify novel markers that may be efficiently targeted for treatment to enhance the prognosis of this highly aggressive subtype.

DLAT serves as the E2 component of the mitochondrial pyruvate dehydrogenase complex (PDC). Prior research has primarily focused on investigating the role and mechanism of DLAT in glycometabolism. The primary role of DLAT in the glycometabolism pathway is to catalyze the oxidative decarboxylation of pyruvate to acetylcholine A.^8^ In a recent investigation conducted by Tsvetkov et al. (2022), DLAT was shown to play a crucial role in a newly discovered mode of cell death, namely cuproptosis, as a lipoylated component involved in cell cuproptosis-related biological processes.^9^ Currently, certain bioinformatics studies are investigating the function and attributes of DLAT in malignancies, demonstrating its potential significance in tumors.^10,11^ Moreover, the roles of DLAT have been experimentally validated in several tumor types, including non-small cell lung cancer,^12^ hepatocellular carcinoma,^13^ clear cell renal cell carcinoma,^14^ and gastric cancer.^15^ Nevertheless, empirical research regarding the role and mechanism of DLAT in BC is lacking.

The Hippo signaling pathway is a tumor suppressor pathway initially identified in Drosophila, playing a key role in regulating tissue homeostasis, organ development, and tumor suppression.^16^ The regulatory role of the Hippo signaling pathway in tumors has been reported in various tumors, such as BC,^17^ liver cancer,^18^ lung cancer,^19^ pancreatic cancer,^20^ and colorectal cancer.^21^ Several studies have confirmed that the Hippo/YAP1 axis plays a vital role in TNBC. In previous studies, we found that elevated DLAT expression is correlated with a poor prognosis in patients with BC.^11,22^ Moreover, among the primary subtypes of BC, DLAT demonstrated comparatively high expression in TNBC. Consequently, we examined the function and mechanism of the DLAT in TNBC. This study revealed that DLAT may promote malignancy in TNBC by interacting with YAP1, facilitating the proliferation, migration, and invasion of TNBC cells. These findings provide a reference for investigation into the role of DLAT in TNBC and for identifying novel targets for the treatment of TNBC.

## 2. Materials and methods

### 2.1. Bioinformatics analysis

UALCAN (https://ualcan.path.uab.edu/analysis.html) was utilized to examine the mRNA levels, protein levels, and survival outcomes associated with DLAT in BC. The Kaplan – Meier plotter (http://kmplot.com/analysis/) was employed to assess the prognostic value of DLAT in patients with BC. The expression correlation data pertaining to DLAT and YAP1 was evaluated using the StarBase database (https://rnasysu.com/encori/).

### 2.2. Cell culture

The human BC cell lines (MDA-MB-231, BT549, MCF-7, and SKBR3) and the human embryonic kidney cell line (HEK293T) were acquired from the Chinese Academy of Sciences (Shanghai, China). The MDA-MB-231, MCF-7, and HEK293T cell lines were cultured in Dulbecco’s Modified Eagle Medium (DMEM, Servicebio) supplemented with 10% fetal bovine serum (FBS, Gibco) and 1% penicillin-streptomycin (P/S, Servicebio). The BT549 cell line was cultured at Roswell Park Memorial Institute-1640 (RPMI-1640, Servicebio) with 10% FBS and 1% P/S. The SKBR3 cell line was cultured in McCoy’s 5A (Procell) with 10% FBS and 1% P/S. All cell lines were maintained at 37°C in a 5% CO2 atmosphere.

### 2.3. Cell transfection

Small interfering RNAs (siRNAs) and negative controls were synthesized by Generay (Shanghai, China). Transfections were performed using Lipo8000™ (Beyotime) following the manufacturer’s instructions. A DLAT overexpression lentivirus and a negative control were purchased from the MiaoLing Plasmid Platform (Shanghai, China). Lentiviral vectors for LV-DLAT and LV-vector were constructed and transfected into MDA-MB-231, BT549, and HEK293T cell lines using the ZORIN Lentiviral Packaging Kit (ZORIN) as per the manufacturer’s instructions to generate stable DLAT-overexpressing cell lines. These stable overexpressing cell lines were selected using 2 μg/mL puromycin (Beyotime).

### 2.4. Immunofluorescence (IF) assay

The antibodies used in the IF assay were as follows: anti-DLAT (A14530, Abclonal) and Alexa Fluor 488 AffiniPure Goat Anti-Rabbit IgG (H+L) (33106ES60, Yeasen). Nuclei were labeled with 4′,6-diamidino-2-phenylindole (DAPI). A fluorescence microscope (Leica DM6B, Germany) was used to capture images of the cells.

### 2.5. RNA extraction and complementary DNA (cDNA) synthesis

Total RNA was extracted from the cells using the EZ-press RNA Purification Kit (B0004D, EZBioscience). The concentration and purity of RNA samples were assessed using a Nanodrop 2000 spectrophotometer (Thermo Fisher Scientific, USA). cDNA was synthesized using the HiScript® II Q RT SuperMix for qPCR (+gDNA wiper) (Vazyme).

### 2.6. Quantitative real-time polymerase chain reaction (qRT-pcr)

qRT-PCR was performed using the Hieff qPCR SYBR Green master mix (Yeasen) on the QuantStudio™ Dx Real-Time PCR Instrument (Applied Biosystems, USA). Data were normalized to GAPDH and quantified using the 2-ΔΔCt method to analyze mRNA expression levels. All primer sequences were synthesized by Generay (Shanghai, China).

### 2.7. MTT assay

TNBC cells transfected with siRNA or plasmid were seeded in 96-well plates at a density of 2000 cells/well. An MTT assay kit (Sigma) was utilized to measure absorbance at 490 nm, following the manufacturer’s instructions. Absorbance was recorded at five detection nodes (24, 48, 72, 96, and 120 h) using a microplate reader (Bio-Tek, USA).

### 2.8. Colony formation assay

Treated cells were seeded into six-well plates at a density of 800 cells/well. The medium was replaced every 3 days, and the cells were incubated at 37°C with 5% CO2 for 7 to 10 days. Subsequently, the cell colonies were fixed in 95% ethanol and stained with 0.01% crystal violet (Yeasen). Ultimately, colonies were enumerated and photographed after washing and drying.

### 2.9. Wound-healing assay

Transfected cells were seeded in six-well plates at 90% confluence. Once the cells had fully adhered, the cell monolayer was scraped using a sterile 200-μL pipette tip. Phosphate-buffered saline (PBS, Yeasen) was used to remove suspended cells, while the monolayers were cultured in a DMEM containing 2% FBS. Wound healing was evaluated under a microscope, and images were captured at the same location at 0, 24, and 48 h time points to observe cell migration. The wound closure rate was calculated as follows: Wound closure rate = (0 h scratch width − 48 h scratch width)/0 h scratch width × 100%.

### 2.10. Transwell assay

For the invasion assay, the treated cells were seeded into the upper chamber of Transwell® permeable supports (Corning Costar Corp., Cambridge) with Matrigel (BD Biosciences) at a density of 3 × 104 cells/chamber. The lower chamber was supplemented with a medium containing 10% FBS, while the upper chamber was filled with a serum-free medium. After 18 h, the cells located on the opposite side of the filter were fixed using 95% ethanol and subsequently stained with 0.01% crystal violet. Representative images were captured under a microscope (Leica Microsystems, Germany), and stained cells were counted in five randomly selected areas.

### 2.11. Subcellular fractionation

Nuclear and cytoplasmic proteins were isolated using the Nuclear and Cytoplasmic Protein Extraction Kit (20126ES50, Yeasen) according to the manufacturer’s instructions. β-actin (AC026, ABclonal) was used as the cytoplasmic control, and histone H3 (A2348, ABclonal) was used as the nuclear control.

### 2.12. Protein extraction and western blot analysis

Total proteins were extracted from cells using RIPA buffer (Beyotime) containing 1 mm PMSF (Beyotime) and a phosphatase inhibitor cocktail C (Beyotime). Protein concentrations were measured using the bicinchoninic acid (BCA) protein assay kit (Beyotime). For each sample, 30–50 μg of protein were separated on 10% sodium dodecyl sulfate-polyacrylamide (SDS-PAGE) gels and then transferred onto nitrocellulose membranes (Beyotime). Afterward, the membranes were blocked with 5% nonfat milk for more than 1 h at 25°C and incubated with primary antibodies at 4°C. The following antibodies were used for western blotting: anti-DLAT (A14530, Abclonal), anti-YAP1 (A1002, Abclonal), anti-p-YAP1 (Ser397) (AP0922, Abclonal), Anti-LATS1/2(DF7517, Affinity), anti-p-LATS1/2 (Ser909/Ser872) (AF8163, Affinity), anti-CTGF (A11456, Abclonal), anti-histone H3 (A2348, Abclonal), anti-β-actin (AC026, Abclonal), anti-IgG (AC005, Abclonal), anti-HA tag (ab236632, Abcam), and anti-FLAG tag (ab236777, Abcam). The immunoblots were scanned using the Amersham Imager 600 (GE, USA).

### 2.13. Co-immunoprecipitation (CoIP) assay

Total proteins used for CoIP were extracted from cells using immunoprecipitation lysis buffer (abs955, Absin) containing 1 mm PMSF (Beyotime) and phosphatase inhibitor cocktail C (Beyotime). CoIP was performed by adding protein A and protein G agarose beads (abs955, Absin), and the final collection of proteins was completed according to the manufacturer’s instructions. Samples were analyzed by western blotting.

### 2.14. Statistical analysis

The data were displayed and analyzed using GraphPad Prism 9.0 (GraphPad Software, CA, USA). Results were presented as mean ± standard deviation (SD) from three independent experiments. Student’s t-test was employed for two-group comparisons, while a two-way ANOVA was performed to assess the results of the MTT assay. A significance level of *p* < .05 was used to determine the statistical significance.

## 3. Results

### 3.1. Expression and characteristics of DLAT in BC

**Figure 1.**
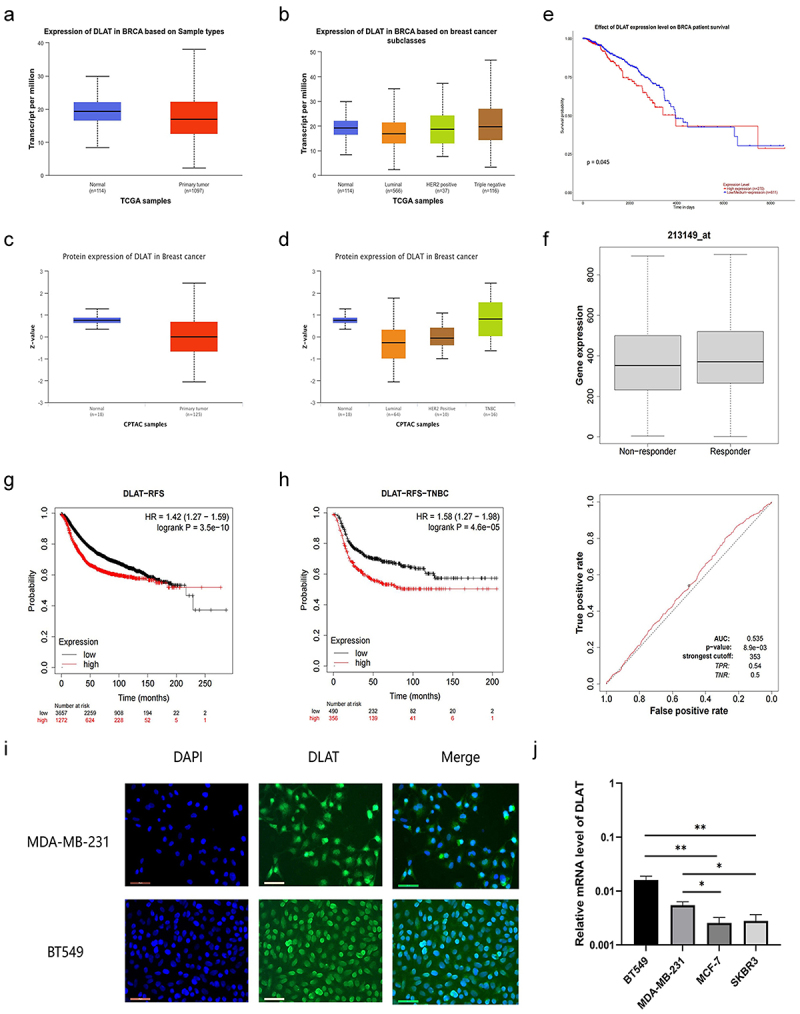
The expression and characteristics of DLAT in BC. (a) DLAT mRNA expression in BC and normal breast tissues from the TCGA database. (b) DLAT mRNA expression in subclasses of BC from the TCGA database. (c) DLAT protein expression in BC and normal breast tissues from the CPTAC database. (d) DLAT protein expression in subclasses of BC from the CPTAC database. (e) Effect of DLAT expression level on BC patient survival from the TCGA database. (f) ROC curve for DLAT expression to diagnose BC in the ROC plot database. (g) RFS curve in DLAT high and low expression BC cases from Kaplan – Meier plotter database. (h) RFS curve in DLAT high and low expression TNBC cases from Kaplan – Meier plotter database. (i) Immunofluorescence of DLAT localization in MDA-MB-231 and BT549 cells. Red, DLAT; blue, DAPI. (j) RT-qPCR analysis of relative expression of DLAT in BT549, MDA-MB-231, MCF-7 and SKBR3 BC cell lines. * represent *p* < .05, ** represent *p* < .01.

An examination of mRNA transcriptome data from BC in The Cancer Genome Atlas (TCGA) revealed that DLAT transcript levels were reduced in tumor tissues compared to normal tissues (Figure 1a). Additional investigation into DLAT expression in different BC subcategories revealed relatively elevated DLAT expression levels in TNBC compared to hormone receptor-positive and HER2-positive BC subtypes (Figure 1b). Similar patterns were observed in the analysis of DLAT protein expression levels in BC using the CPTAC database (Figures 1c,d). Analysis of survival data from TCGA database indicated that higher DLAT expression was associated with poorer overall survival (OS) outcomes (Figure 1e). A receiver operating characteristic (ROC) curve was plotted to evaluate DLAT as a potential biomarker for the diagnosis of BC. The results from the ROCplot database indicated that, despite a *p* value of <0.001, the AUC was 0.535, suggesting that the DLAT may not be a suitable diagnostic marker for BC (Figure 1f). Further analysis using the Kaplan – Meier plotter database showed that patients with BC exhibiting higher DLAT expression displayed shorter recurrence-free survival (RFS) (Figure 1g). This trend was more pronounced in patients with further differentiated TNBC (Figure 1h). Immunofluorescence analysis of MDA-MB-231 and BT549 cell lines revealed the presence of DLAT in both the cytoplasm and nucleus (Figure 1i). qRT-PCR analysis of cell lines was consistent with TCGA transcriptome data, showing relatively high DLAT expression in the TNBC subtype cell line of BC (Figure 1j). These results indicate that higher DLAT expression levels are associated with a worse prognosis in patients with BC, and DLAT is relatively highly expressed in the TNBC subtype, suggesting that DLAT may have a more pronounced impact on the malignant behavior of tumors in patients with TNBC.

### 3.2. DLAT contributes to the proliferation of TNBC cells

**Figure 2.**
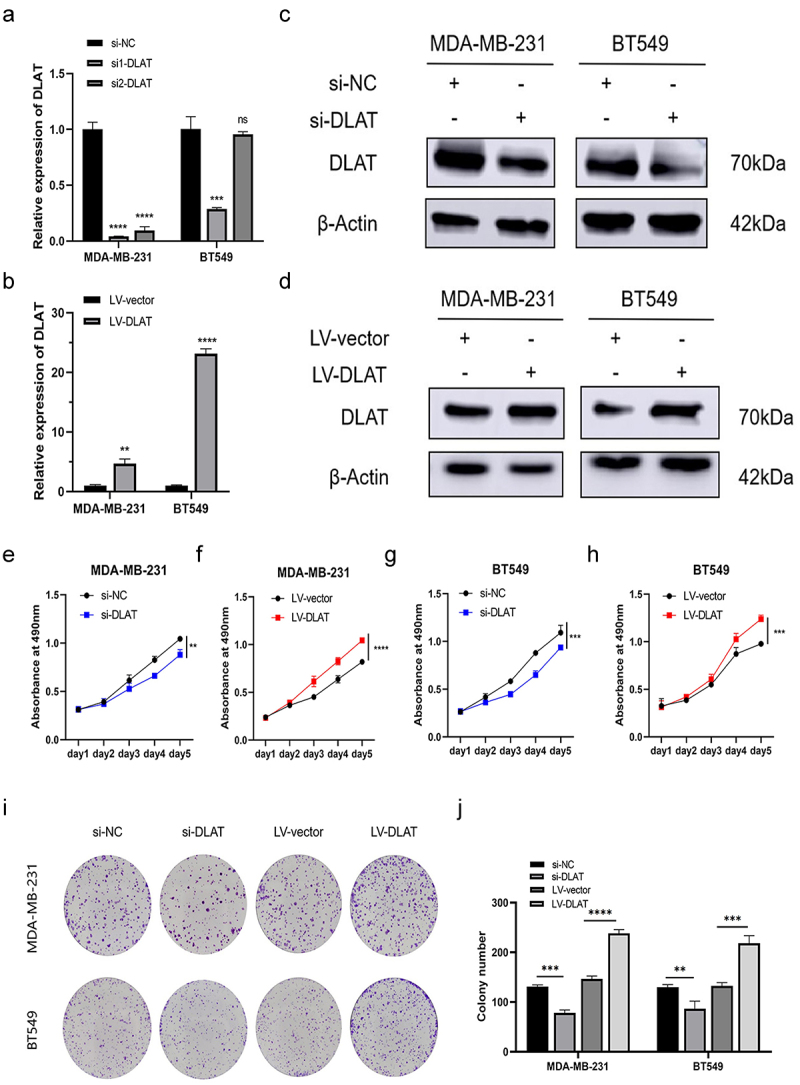
DLAT contributes to proliferation in TNBC cells. (a) RT-qPCR analysis of knockdown efficiency of si1-dlat, si2-dlat and negative control (si-nc) in MDA-MB-231 and BT549. (b) RT-qPCR analysis of overexpression efficiency of LV-DLAT and negative control (lv-vector) in MDA-MB-231 and BT549. (c) Western blot analysis of knockdown efficiency of si1-dlat, si2-dlat and negative control (NC) in MDA-MB-231 and BT549. (d) Western blot analysis of overexpression efficiency of LV-DLAT and negative control (lv-vector) in MDA-MB-231 and BT549. (e-h) the effects of si-DLAT and LV-DLAT on the proliferation of MDA-MB-231 and BT549 cells were detected by MTT assay. (i, j) the effects of si-DLAT and LV-DLAT on the proliferation of MDA-MB-231 and BT549 cells were detected by colony formation assays. ns represents no statistics differences, ** represent *p* < .01, *** represent *p* < .001, **** represent *p* < .0001.

To further investigate the biological function of DLAT in TNBC, we knocked down DLAT by transfecting DLAT siRNA and negative control siRNA into the TNBC cell lines MDA-MB-231 and BT549. We constructed cell lines stably overexpressing DLAT and corresponding negative control cell lines using lentiviral vectors. The interference efficiency of si1-DLAT and si2-DLAT in the two TNBC cell lines was validated by qRT-PCR. si1-DLAT, which exhibited a more consistent interference efficiency, was ultimately chosen for further research (Figure 2a). The overexpression efficiency of DLAT in stably transfected cells was also assessed using qRT-PCR (Figure 2b). DLAT protein levels in MDA-MB-231 and BT549 cell lines after DLAT knockdown and overexpression were detected via western blotting (Figures 2c,d). MTT and colony formation assays demonstrated that the downregulation of DLAT inhibited the proliferation of MDA-MB-231 and BT549 cells, while the overexpression of DLAT had the opposite effect (Figures 2e–j). Thus, DLAT contributes to the proliferation of TNBC cells.

### 3.3. DLAT promotes migration and invasion of TNBC cells

**Figure 3.**
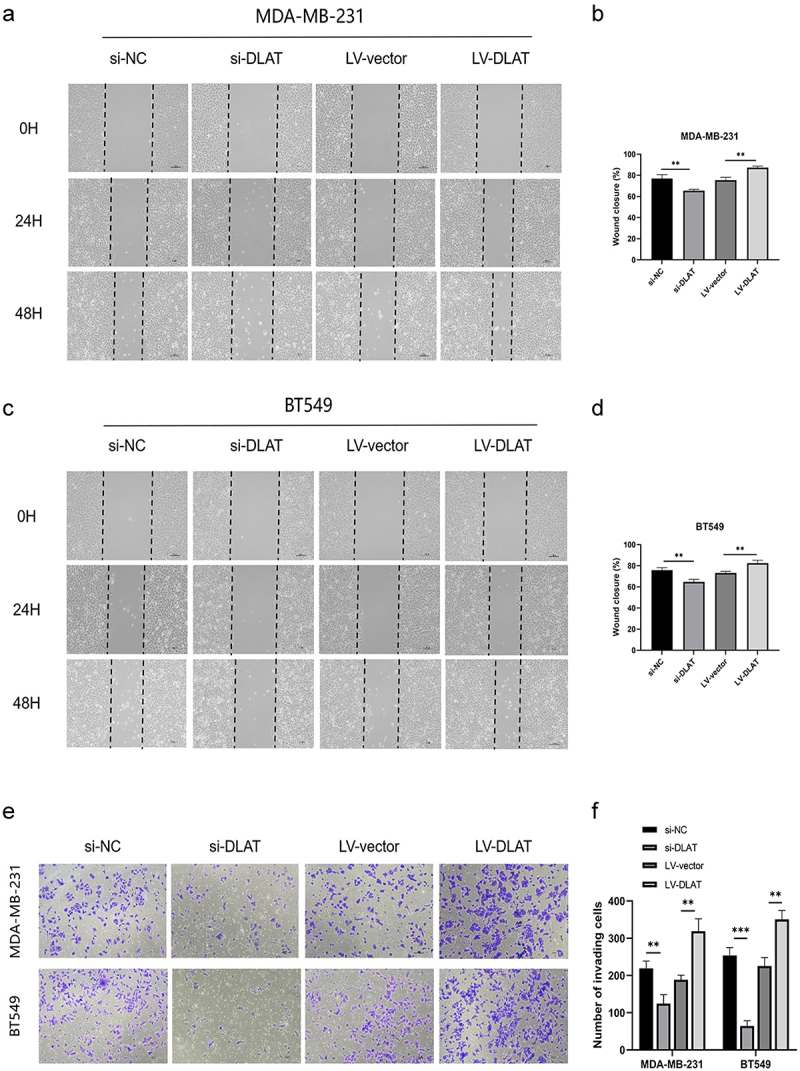
DLAT promotes migration and invasion of TNBC cells. (a, b) effect of si-DLAT and LV-DLAT on migration abilities in MDA-MB-231 cells by wound-healing assays. (c, d) effect of si-DLAT and LV-DLAT on migration abilities in BT549 cells by wound-healing assays. (e, f) effect of si-DLAT and LV-DLAT on invasion abilities in MDA-MB-231 and BT549 cells by transwell invasion assays. ** represent *p* < .01, *** represent *p* < .001.

The wound-healing assay was employed to observe and record the migration of MDA-MB-231 and BT549 cells at three time points (0, 24, and 48 h) following the knockdown and overexpression of DLAT. Over time, the loss of function of the DLAT demonstrated an inhibitory effect on cell migration, whereas the gain of function of DLAT promoted cell migration (Figures 3a–d). The effect on cell invasion ability was validated through a Transwell assay. The expression of DLAT was significantly proportional to the invasive ability of TNBC cells (Figure 3e,f). In line with the pro-proliferative effects of DLAT in TNBC cell lines, DLAT generally exhibited oncogenic effects in TNBC cells.

### 3.4. DLAT promotes TNBC progression by activating YAP1 signaling

**Figure 4.**
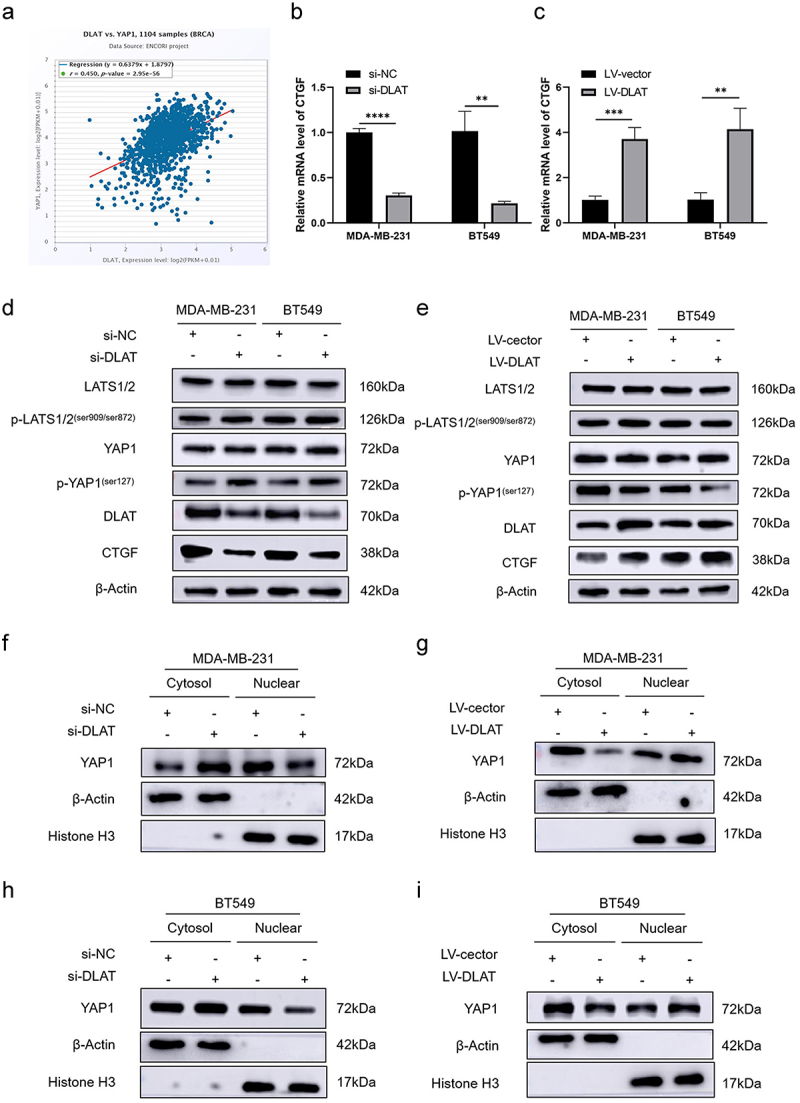
DLAT promoted TNBC progression via activating YAP1 signaling. (a) DLAT expression positively correlates with YAP1 expression from the starBase database. (b) RT-qPCR analysis of the mRNA levels of CTGF in si-dlat and si-nc treated cells. (c) RT-qPCR analysis of the mRNA levels of CTGF in LV-DLAT and LV-vector treated cells. (d) Western blot analysis of expression level of DLAT, total YAP1 and p-YAP1 in si-dlat and si-nc treated cells. (e) Western blot analysis of expression level of total LATS1/2, p-LATS1/2, DLAT, total YAP1, p-YAP1 and CTGF in LV-DLAT and LV-vector treated cells. (f-i) Nuclear-cytoplasm separation assay indicated that knockdown and overexpression of DLAT regulated YAP1 translocation between nuclear and cytoplasm in MDA-MB-231 and BT549 cells. ** represent *p* < .01, *** represent *p* < .001, **** represent *p* < .0001.

We found that YAP1 potentially interacts with the DLAT, as evidenced by the Starbase database (Figure 4a). YAP1 plays a crucial role as an effector in the Hippo signaling pathway. The entry-exit nuclear behavior of YAP1 is regulated by the Hippo pathway via the MST1/2-LATS1/2-YAP1 phosphorylation cascade, subsequently affecting the expression of downstream oncogenes, including CTGF. qRT-PCR showed that the knockdown of DLAT inhibited the expression of CTGF, while the overexpression of DLAT activated the expression of CTGF (Figures 4b,c). Western blot analysis revealed that the phosphorylation level of YAP1 increased and the expression level of CTGF decreased following the knockdown of DLAT in the MDA-MB-231 and BT549 cell lines. Conversely, the overexpression of DLAT impeded the phosphorylation of YAP1, followed by the upregulation of CTGF expression (Figures 4d,e). Intriguingly, the knockdown and overexpression of DLAT neither affect the expression of the upstream key factor LATS1/2 of YAP1 nor its phosphorylation, suggesting that the effect of DLAT on the phosphorylation of YAP1 is not mediated by LATS1/2. To investigate the potential impact of DLAT expression on the subcellular localization of YAP1, we conducted a plasmo-nuclear separation test to isolate cytoplasmic and nuclear proteins for subsequent western blot analysis. When the DLAT was silenced, the YAP1 levels decreased in the nucleus and correspondingly increased in the cytoplasm. The overexpression of DLAT promoted the nuclear entry of YAP1 (Figures 4f–i). These results suggest that DLAT can affect the subcellular distribution of YAP1 by modulating its phosphorylation, thereby impacting the expression of downstream genes and ultimately influencing the biological behavior of TNBC.

### 3.5. Protein–protein interactions were observed between DLAT and YAP1

**Figure 5.**
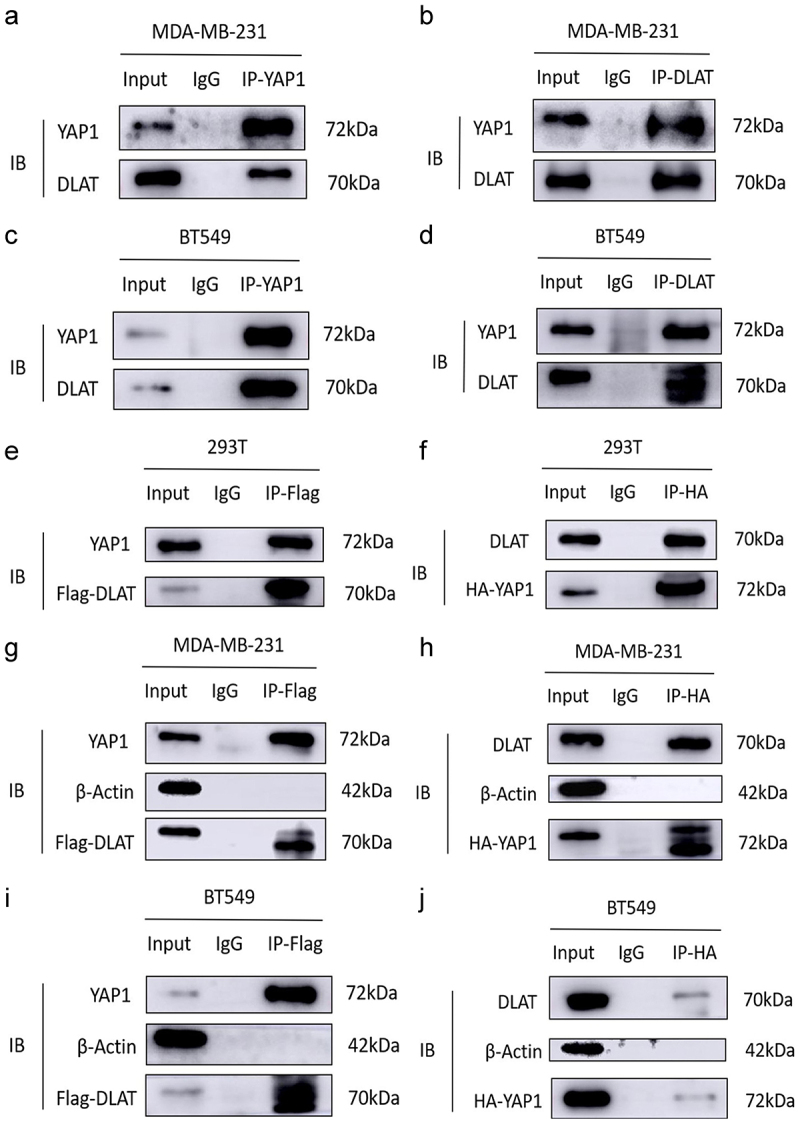
Protein–protein interactions were found between DLAT and YAP1. (a-d) CoIP assays showed the endogenous interaction between DLAT and YAP1 in MDA-MB-231 and BT549 cells. (e, f) CoIP assays showed the exogenous interaction between DLAT and YAP1 in HEK293T cells. (g-j) CoIP assays showed the exogenous interaction between DLAT and YAP1 in MDA-MB-231 and BT549 cells.

Both endogenous and exogenous CoIP assays were performed to further validate the molecular mechanism of DLAT affecting YAP1. First, the endogenous CoIP validated the direct interaction between DLAT and YAP1 (Figures 5a–d). Next, we constructed a FLAG-tagged DLAT overexpression plasmid and an HA-tagged YAP1 overexpression plasmid for exogenous validation. HEK293T, MDA-MB-231, and BT549 cell lines were co-transfected with the aforementioned plasmids. CoIP/Western blotting analysis showed that DLAT interacted with YAP1 through protein – protein interaction (Figures 5e–j). Overall, DLAT exerts its regulatory role by directly binding to YAP1.

### 3.6. DLAT promotes the malignant behavior of TNBC in a YAP1-dependent pathway

**Figure 6.**
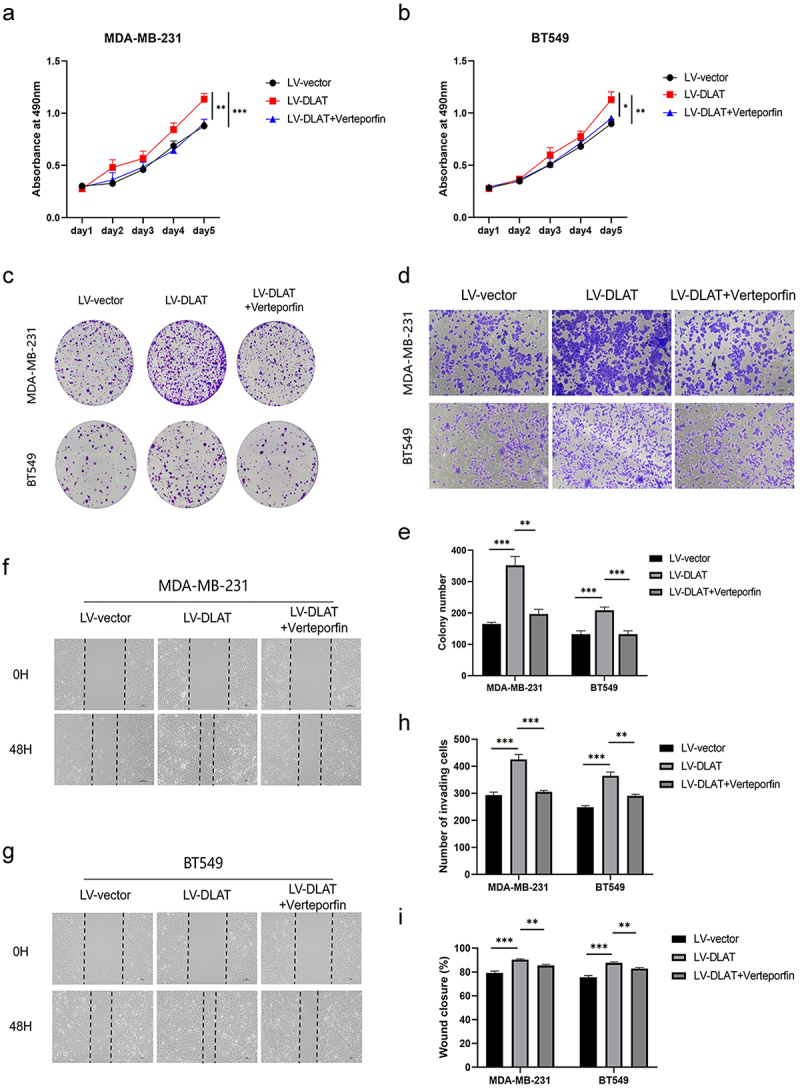
DLAT promotes the malignant behavior of TNBC in a YAP1-dependent pathway. (a, b) effects of LV-DLAT and Verteporfin co-treated on the proliferation of MDA-MB-231 and BT549 cells were detected by MTT assay. (c, e) effects of LV-DLAT and Verteporfin co-treated on the proliferation of MDA-MB-231 and BT549 cells were detected by colony formation assays. (d, h) effect of LV-DLAT and Verteporfin co-treated on invasion abilities in MDA-MB-231 and BT549 cells by transwell invasion assays. (f, g, i) effect of LV-DLAT and Verteporfin co-treated on migration abilities in MDA-MB-231 and BT549 cells by wound-healing assays. * represent *p* < .05, ** represent *p* < .01, *** represent *p* < .001.

To validate whether DLAT exerts its effects on TNBC in a YAP1-dependent manner, we conducted rescue experiments. Verteporfin functions as a micromolecular inhibitor of YAP1, effectively impeding its transcriptional activity by binding to YAP and preventing its entry into the nucleus. Verteporfin was used to inhibit the expression of YAP1 in DLAT-overexpressing cell lines. MTT and colony formation assays demonstrated that YAP1 inhibition reversed the pro-proliferative behavior of TNBC induced by DLAT overexpression (Figures 6a–c,e). Moreover, the YAP1 inhibition abolished the promotion of TNBC migration and invasion caused by DLAT upregulation (Figures 6d,f–i). Taken together, these results suggest that the cancer-promoting behavior of DLAT in TNBC is contingent upon YAP1 activation.

## 4. Discussion

TNBC is generally regarded as a clinically aggressive subtype that typically occurs in young women and requires auxiliary chemotherapy to improve survival.^23^ Compared with hormone receptor-positive subtypes of BC, TNBC exhibits a different recurrence pattern. More than one-third of patients with TNBC exhibit the potential to develop distant metastasis within 3 years of diagnosis and are more likely to present distant metastases in the brain and lungs.^24;25^ At present, conventional chemotherapy with cytotoxic drugs is commonly employed for patients with TNBC; however, adverse reactions, such as bone marrow suppression and neurotoxicity, are prone to occur, leading to intolerance in patients.^26;27^ Given the limited efficacy of chemotherapy, investigating targeted approaches for TNBC is imperative to enhance clinical outcomes for patients.

In this study, we found that DLAT was significantly associated with the development and progression of TNBC. DLAT expression was lower in BC tumor tissues than in normal tissues at both the transcriptional and protein levels. However, the high DLAT expression was associated with poorer survival. Among the four major subtypes of BC, DLAT expression is relatively higher in TNBC, suggesting that it may dominate more pronounced tumor malignancy in this subtype. Investigation into cell proliferation, migration, and invasion in TNBC cell lines revealed that upregulation of DLAT exhibited tumor-promoting characteristics, whereas downregulation of DLAT demonstrated tumor-suppressor functions. This suggests that DLAT may function as an oncogene in the biological processes underlying TNBC.

The Hippo signaling pathway is an evolutionarily conserved pathway for growth control and tumor suppression.^28^ As a pivotal signaling cascade governing cell proliferation, differentiation, and survival, its frequent dysregulation in diverse malignancies is intricately associated with tumorigenesis and tumor progression.^29^ The primary components of the Hippo signaling pathway include MST1/2, LATS1/2, SAV1, and MOB1A/B, which function by phosphorylating YAP1, the main effector of the Hippo pathway.^30^ YAP1 shuttles between the cytoplasm and nucleus in a regulated manner.^31^ When the Hippo signaling pathway is activated, it phosphorylates YAP1 through a series of phosphorylation reactions, causing it to bind to the 14-3-3 protein, accumulate in the cytoplasm, and accelerate its ubiquitination and degradation, thus inhibiting YAP1-mediated transcription.^32^ Conversely, when the Hippo signaling pathway is inactivated, the YAP1 nuclear translocation increases, allowing it to interact with TEAD1–4 to activate gene transcription, promote tissue growth, and inhibit apoptosis.^33,34^ A notable correlation has been observed between elevated YAP1 expression levels and the presence of hormone receptor negativity, tumor aggressiveness, and worse clinical outcomes.^35,36^ Moreover, high expression of YAP1 in patients with TNBC receiving neoadjuvant chemotherapy may affect the pathological complete response rate and long-term survival while also increasing the likelihood of recurrence.^37^ This suggests that the Hippo/YAP1 axis plays a vital role in TNBC. Based on gene co-expression predictions in the database, this study found that YAP1 potentially interacts with DLAT. We validated that DLAT affects the translocation of YAP1 to the nucleus, which further regulates the expression of CTGF, a downstream oncogene of YAP1, and ultimately promotes the progression of TNBC. The CoIP assay validated that DLAT directly interacts with YAP1 to regulate its expression. Rescue experiments further demonstrated that verteporfin, a micromolecular inhibitor targeting YAP1, reverses the effects of DLAT overexpression on TNBC proliferation, migration, and invasion.

However, there are certain limitations to this study. Although current studies effectively elucidate the role and partial mechanism of DLAT in TNBC, there is still a lack of clinical sample verification, and some experimental designs may have confounding factors given the existing technology. Therefore, future studies will involve collecting clinical breast cancer samples to detect DLAT expression. To explore novel YAP1-specific phosphorylation inhibitors for the control experiment of plasmo-nuclear separation test to reduce confounding factors and further optimization of experimental design for deeper exploration and so on.

## 5. Conclusions

Our study is the first to reveal the tumor-promoting role of DLAT in TNBC. We validated that DLAT directly binds to YAP1, inhibiting its phosphorylation and promoting its nuclear translocation, thereby promoting TNBC progression. These findings provide novel insights into the molecular mechanisms underlying TNBC progression and may offer a promising strategy for its treatment.

## Data Availability

Data will be made available on request.

## List of abbreviations

BC breast cancer

TNBC triple-negative breast cancer

DLAT Dihydrolipoamide S-acetyltransferase

CoIP Coimmunoprecipitation

ER estrogen receptor

PR progesterone receptor

HER2 human epidermal growth factor 2 receptor
